# PrEP-Related Medical, Structural and Institutional Mistrust among a Socioeconomically Diverse Sample of Black, Latine, Asian, and White Young Sexual Minority Men: Lived Experiences of Intersectional Inequity

**DOI:** 10.1007/s10461-025-04882-w

**Published:** 2025-10-06

**Authors:** Jessica Jaiswal, Marybec Griffin, Steven Meanley, Jerel M. Ezell, Yahya Alnashri, Kevin Hascher, Benjamin Grin, Perry N. Halkitis

**Affiliations:** 1 Department of Family and Community Medicine, Heersink School of Medicine, University of Alabama at Birmingham, Birmingham, AL USA; 2 Center for Interdisciplinary Research on AIDS, Yale University, New Haven, CT USA; 3 Department of Health Behavior, Society & Policy, Center for Health, Identity, Behavior & Prevention Studies, Rutgers University, New Brunswick, NJ USA; 4 Department of Family and Community Health, University of Pennsylvania School of Nursing, Philadelphia, PA USA; 5 Community Health Sciences, School of Public Health, University of California Berkeley, Berkeley, CA USA; 6 Berkeley Center for Cultural Humility, University of California Berkeley, Berkeley, CA USA; 7 Department of Health Policy and Organization, School of Public Health, University of Alabama at Birmingham, Birmingham, AL USA; 8 Feinberg School of Medicine, Northwestern University, Evanston, IL USA; 9 Department of Primary Care, Kansas City University College of Osteopathic Medicine, Kansas City, MO USA; 10 Center for Health, Identity, Behavior & Prevention Studies, Rutgers University, New Brunswick, NJ USA; 11 Department of Biostatistics & Social and Behavioral Health Sciences, Rutgers School of Public Health, Rutgers University, New Brunswick, NJ USA

**Keywords:** Mistrust, Distrust, HIV, PrEP, Sexual minority, Medication, Desconfianza, Recelo, VIH, PrEP, Minoría sexual, Medicación

## Abstract

Mistrust among marginalized populations has been shown to negatively impact health behaviors and outcomes. Young sexual minority men (YSMM) experience many health inequities, including those related to HIV. Understanding how YSMM think about and experience medical, structural, and institutional mistrust is critical to effectively promoting engagement in preventive services like pre-exposure prophylaxis (PrEP). The study recruited 43 YSMM (ages 25–27) from diverse racial, ethnic, and socioeconomic backgrounds in New York City. Semi-structured interviews were conducted from July- November 2018 and focused on sexual health, HIV-related beliefs, PrEP, and experiences with healthcare systems and providers. Three main themes were identified: (1) concerns regarding PrEP as a medication, including its perceived novelty, potential side effects, and the perceived lack of a compelling reason to take preventive medication; (2) ethical and philosophical apprehensions around perceived U.S. government-pharmaceutical collusion, and (3) PrEP and healthcare providers are not necessarily perceived as nefarious; mistrust can coexist with support for PrEP. Although most participants expressed some level of government-pharmaceutical mistrust, such mistrust did not necessarily dissuade them from supporting or even taking PrEP. While most participants did not consider healthcare providers as actors in government-pharmaceutical collusion, clinicians can play an essential role in addressing patients’ concerns and building trust. Clinicians should endeavor to make space for open, non-judgmental conversations not only about sexual behavior, but also patients’ experiences of discrimination and socioeconomic exclusion. Finally, structural interventions must seek to address societal and institutional inequities to undo harm and earn trust.

## RESUMEN

Se ha demostrado que la desconfianza entre poblaciones marginadas impacta negativamente en los comportamientos y resultados relacionados con la salud. Los hombres jóvenes pertenecientes a minorías sexuales (HJMS) experimentan numerosas inequidades en salud, incluidas aquellas relacionadas con el VIH. Comprender cómo los HJMS piensan y experimentan la desconfianza médica, estructural e institucional es fundamental para promover de manera efectiva la participación en servicios preventivos como la profilaxis preexposición (PrEP). El estudio reclutó a 43 HJMS (de entre 25 y 27 años) provenientes de diversos contextos raciales, étnicos y socioeconómicos en la ciudad de Nueva York. Se realizaron entrevistas semiestructuradas entre julio y noviembre de 2018, centradas en la salud sexual, creencias relacionadas con el VIH, la PrEP y experiencias con los sistemas de salud y sus proveedores. Se identificaron tres temas principales: (1) preocupaciones en torno a la PrEP como medicamento, incluida su novedad percibida, los posibles efectos secundarios y la falta percibida de una razón convincente para tomar un medicamento preventivo; (2) aprensiones éticas y filosóficas respecto a una supuesta colusión entre el gobierno de los Estados Unidos y la industria farmacéutica; y (3) la PrEP y los proveedores de salud no son necesariamente percibidos como entidades malintencionadas; la desconfianza puede coexistir con el apoyo a la PrEP. Aunque la mayoría de los participantes expresó cierto nivel de desconfianza hacia la relación gobierno-industria farmacéutica, dicha desconfianza no necesariamente los disuadió de apoyar o incluso usar la PrEP. Aunque la mayoría no consideró a los proveedores de salud como actores en la colusión entre el gobierno y la industria farmacéutica, los profesionales clínicos pueden desempeñar un papel esencial en la atención a las inquietudes de los pacientes y en la construcción de confianza. Los clínicos deben procurar generar espacios para conversaciones abiertas y sin juicios, no solo sobre el comportamiento sexual, sino también sobre las experiencias de discriminación y exclusión socioeconómica de los pacientes. Finalmente, las intervenciones estructurales deben orientarse a abordar las inequidades sociales e institucionales con el fin de reparar daños y generar confianza.

## Introduction

A myriad of literature in public health and medicine has long documented the relationship between medical mistrust and suboptimal health behaviors and outcomes among historically marginalized populations [1]. Despite inconsistent operationalization [1, 2], medical mistrust is often defined as the lack of trust in healthcare providers [2]; however, mistrust and lack of trust are not synonymous. Mistrust suggests active suspicion of harm rather than merely the absence of trust [3]. Moreover, health-related mistrust is broader in scope than just concerns about healthcare providers [1], encompassing structural and institutional mistrust that shape the environments in which people make decisions about their health [4]. 

In the U.S., research evaluating medical mistrust in the context of HIV/AIDS has focused largely on Black communities [5–8]. Prior studies have largely found that medical mistrust negatively affects health behaviors and outcomes, such as lower CD4 cell counts, higher HIV viral loads [9], poor adherence to Antiretroviral therapy (ART), and care engagement [6, 10, 11]. Although the literature largely points to key historical events such as the Tuskegee experiments as main drivers of mistrust, it is critical to acknowledge the contributions of ongoing social and economic inequities that continue to place minoritized communities at risk for health disparities [12]. Pervasive structural racism and white supremacy (e.g., residential segregation, economic disenfranchisement, mass incarceration, etc.) [13, 14], stigma, homophobia and transphobia [4], systematic lack of economic opportunities [15], and ongoing policies that reinforce inequity (e.g., lack of Medicaid expansion) [16] work in tandem to thwart access to comprehensive prevention and management tools.

Beyond medical and health-related mistrust specifically, structural mistrust, while under-explored, warrants further exploration. Structural and institutional mistrust refers to suspicion or skepticism toward systems of power, often held by groups with less social and economic power, based on perceptions and experiences of ill intent or inequitable treatment [4, 17–19]. Such notions include that the government and/or the pharmaceutical industry is deliberately withholding a cure for HIV to continue to profit off ART, and that the government/Big Pharma is deliberately withholding a cure for HIV in order to continue to profit off ART [19, 20]. For example, in a 2016 national survey, 31% of Black adults believed HIV was human-made, 40% thought a cure was being withheld by the government, 33% believed HIV medications are poison, and only 18% trusted government health agencies [21]. Although personal resilience can enhance engagement in HIV care among Black and Latine youth, its protective effect is significantly weakened in contexts of high system-level mistrust [22]. Less is known about structural and institutional mistrust in the context of HIV prevention, as most research has focused on populations with HIV [23, 24]. 

Young sexual minority men (YSMM), defined here as men in their early to mid-20s, are at higher risk for contracting HIV due to a constellation of interrelated biopsychosocial factors [25]. In prior studies with samples of YSMM, researchers have provided robust findings that exhibit inverse associations between medical mistrust and general [8, 26], HIV-related [10], and sexual health care engagement [8, 27–31]. However, many of these studies, largely quantitative, have not accounted for the multi-level nature in which medical mistrust may arise, inadvertently conflating attitudes toward medical systems, health care providers, and HIV-related medications in a single concept. Additionally, these studies have not consistently applied an intersectional lens, operationalizing challenges in these populations in ways that do not account for multiple dimensions of the individual’s identity (e.g., race, ethnicity, gender expression, age, sexual orientation, etc.). Disparate beliefs about various dimensions of health care and society more broadly may contribute to how individuals placed at structural risk for HIV engage in prevention services. Identifying the salient areas of mistrust held by YSMM is warranted and critical to implementing focused interventions that promote routine engagement in HIV prevention services and PrEP adoption. Given that the uptake of biomedical HIV prevention requires some level of trust in and acceptance of pharmaceutical science, prescribers, and manufacturers, it is important to understand how YSMM think about and navigate healthcare systems, institutions, and the pharmaceutical industry.

## Methods

### Study Sample

The Health-Related Beliefs Study was a mixed methods study designed to explore HIV-related beliefs and experiences with sexual health care among YSMM. This sub-study was nested within the P18 Study, a prospective cohort study that examined HIV, substance use, and mental health in a socio-demographically diverse sample of emerging Young adult SMM and transgender women in New York City. Briefly, eligible participants must have been assigned male at birth, HIV-negative, 22–23 years old at the time of enrollment, residing in NYC, and reported recent sexual activity with a man. Parent study participants attended biannual study visits for three years, where they received HIV/STI testing and completed computer-based and interviewer-administered assessments. Further details are available in previous publications [32]. For recruitment for the sub-study presented here, participants were approached in-person during their regularly scheduled parent study data collection appointments or contacted via their preferred method of communication (e.g., phone, email). PrEP use was not part of inclusion/exclusion criteria. Participants quoted in this analysis were 25–27 years old at the time of data collection.

### Data Collection

All HIV-negative parent study participants were invited to participate in the Health-Related Beliefs Study. Qualitative methods enable participants to attribute meaning to their lived experiences and are especially useful when exploring sensitive or stigmatized topics [33]. In-depth qualitative interviews were conducted from July–November 2018 by research staff trained in qualitative interviewing. Interviews took place in-person in an office at the study team’s academic sexual and gender minority health-focused research center.

The research team utilized an intersectional framework [34] and a socioecological lens to inform the study design and data collection instruments. An intersectional approach renders visible how overlapping social and economic structures, such as classism, structural racism, and homophobia, shape people’s daily lived experiences. The qualitative interview guide explored sexual health, HIV, daily oral pre-exposure prophylaxis (PrEP), and experiences with healthcare providers and healthcare systems. Interview length ranged from 30 to 75 min, with most interviews lasting approximately one hour. All participants provided written informed consent, and all activities were approved by the NYU Institutional Review Board.

### Data Analysis

All interviews were audio recorded and professionally transcribed. To ensure data integrity, the research team systematically developed a coding scheme, applied it to the data, and used it to discern patterns, themes, and subcategories. A codebook was developed by the lead and fourth author, and coding was completed by the lead author and three research assistants. As with the interview guide, codebook development was informed by an intersectional framework to help illuminate the interplay of participants’ multiple social identities and structural contexts, and to capture the complexity of their daily lived experiences. The iterative development of this tier-organized tool aided the research team in generating umbrella codes and more specific codes, i.e., subcodes. All research team members contributed to refining the codebook, thereby increasing trustworthiness in the conclusions. Upon completion of coding, codes and subcodes were organized, extracted, and re-examined to identify relationships and themes. Analyses were closely reviewed by the entire research team to discuss and resolve differences in interpretation. During the analysis phase, the team used multiple techniques to establish trustworthiness, including credibility and confirmability. For example, the authors utilized peer debriefing and periodic external audits with researchers not involved in data collection [33]. All qualitative data were organized using Atlas.ti 8. All participants were assigned pseudonyms to protect their confidentiality.

## Results

In total, 43 individuals participated in the interviews, with sociodemographic characteristics summarized in Table 1. Participants were all 25–27 years old and most held a bachelor’s or graduate degree with an annual income above $25,000. In terms of daily oral PrEP use, (58.1%) had never used PrEP, (23.3%) had prior PrEP use, and (18.6%) were currently using PrEP. The sample was predominantly gay (79.1%), with smaller proportions identifying as bisexual (11.6%), queer (7.0%), and pansexual (2.3%).

**Table 1:** Participant sample characteristics (*n* = 43)

	*n* (%)
*Age* (Mean, SD, range)	25.86 (0.74), 25–27
*Race/Ethnicity*	
Asian	9 (20.9)
Black	11 (25.6)
Hispanic/Latine	12 (27.9)
White	11 (25.6)
*Sexual Orientation*	
Gay	34 (79.1)
Bisexual	5 (11.6)
Queer	3 (7.0)
Pansexual	1 (2.3)
*Oral Daily PrEP Status*	
Never used PrEP	25 (58.1)
Prior PrEP use	10 (23.3)
Currently using PrEP	8 (18.6)
*Education*	
High school/GED or less	9 (20.9)
Some college	8 (18.6)
Bachelor’s or graduate degree	26 (60.5)
*Total annual income*,* past year*	
<$15,000	8 (18.6)
$15,000–24,999	9 (20.9)
$25,000–44,999	14 (32.6)
*≥*$45,000	10 (23.3)
Missing	2 (4.7)

### Qualitative Themes

Three main themes emerged from the data. First, participants shared their practical concerns related to PrEP as a medication, particularly around its perceived newness and potential side effects. For many, these concerns outweighed the argument for prevention, which was not compelling. Second, and more broadly, many participants expressed ethical and philosophical concerns about both the federal government and the pharmaceutical industry, including government-pharmaceutical collusion and unrestrained greed on the part of “Big Pharma” and the government that prioritized profits over the needs of ordinary people. Finally, although most participants expressed perceptions of government-pharmaceutical collusion, many participants did not necessarily include healthcare providers and PrEP as part of this collusion narrative. While a minority of participants did express mistrust of healthcare providers specifically as part of the collusion scheme, most did not, with some expressing uncertainty or ambivalence. Notably, most participants were still in support of PrEP, either generally for their community, or for themselves personally.

### Theme 1: Practical Concerns Around PrEP as a Medication

Although largely in support of PrEP access and use, many participants expressed practical concerns around PrEP as a medication, including worries about its perceived newness, potential side effects, and taking a pill daily for prevention. Two subthemes were identified that exemplify how participants grappled with their perceptions and experiences around PrEP: (a) Perceived newness and side effect concerns, and (b) prevention is not compelling.

#### Subtheme 1a: Perceived Newness and Side Effect Concerns

Many participants perceived PrEP as a “new” medication without a proven history of safety. Carlos, quoted below, also expressed his concerns about future unknown side effects:

*It’s a brand-new product. I don’t know how long they’ve been testing it behind closed curtains*,* I don’t know how long people have been on it*,* but more importantly to me*,* I don’t know what side effects are being brought about this. I don’t know if people are gonna have kidney failures 10 years from now… I’m sure that*,* with everything*,* they have the common side effects that they call “common”*,* and at the very end of the commercial*,* they start ranting a whole bunch of side effects that they say really quick*,* for people to just not hear.* (Carlos, Latine, 25, Never used PrEP)

Below, Oliver expresses that he is uncertain if taking PrEP, and thus risking unknown future side effects, would be “worth [it]” given his perception of PrEP having insufficient long-term data:

*For me at least it’s always been – there’s definitely been hesitation because it’s a relatively newer drug… no one’s been on this for 40 years yet. We don’t know the super long-term effects. I feel like when I read about it*,* I sometimes will focus on the bone density or whatever*,* the kind of possible side effects that some people have*,* but just in general it’s something that I would only be using as an extra backup basically that I don’t know that I feel like would be worth any potential side effects if it’s just a backup essentially.* (Oliver, White Non-Hispanic, 25, Never used PrEP)

#### Subtheme 1b: Prevention is not Compelling

Many participants expressed that prevention was not a compelling reason to take PrEP. As most participants were in their early to mid-20s, this reticence about utilizing “unnecessary” medications perhaps reflects their self-perceptions of being young and generally healthy. Even so, the lack of compelling appeal around PrEP seemed to be underscored by underlying concerns, or perhaps suspicion, of the pharmaceutical industry.

Ethan felt that taking PrEP did not align with his values around maintaining his health:

*I just feel like [PrEP is] a sign of needing pharmaceuticals to go about your daily life. And I try to avoid that –… I just try to eat well and work out every day. And that’s just my way of staying healthy. And I’d like that to be it until one day I will need something down the line to keep me healthy.* (Ethan, White Hispanic, 26, Never used PrEP)

Wes, quoted below, echoed earlier participants regarding side effect concerns and his perception that PrEP has not been studied long enough. Thus, he did not feel PrEP, as a preventive medication related to sexual health, was necessary because having sex was “not a necessity for” him:

*So*,* what would make me feel really comfortable about taking PrEP really is I feel like – research… I don’t want to be 80 years old and having complication of this pill I’ve taken for not just having sex… I’m not really trying to put anything into my body that I don’t necessarily need to in order to protect me from something that’s not a necessity… I feel like I can benefit from PrEP*,* theoretically*,* but I just don’t know if there’s enough information for me to make that decision* (Wes, Black Non-Hispanic, 26, Never used PrEP).

### Theme 2: Ethical Concerns about the Pharmaceutical Industry and Government-Pharmaceutical Collusion

Many participants shared their perceptions of the U.S. government’s role in producing and sustaining pervasive social and economic inequality. Three subthemes were identified: (a) The U.S. is a fundamentally broken society due to capitalism, classism, and structural racism; (b) HIV cure/origin beliefs reveal the intersectional nature of structural inequity, and (3) PrEP and healthcare providers are not necessarily part of government-pharmaceutical collusion.

#### Subtheme 2a. The U.S.’s Capitalist System and Entrenched Classism Prioritize Profits Over the Wellbeing of Ordinary People, Particularly within the Healthcare System

Many participants emphasized that the government does not adequately take care of this country’s inhabitants, highlighting that the focus is on capitalism and profits, which often translates into not caring for marginalized people and, in fact, further marginalizing them. Many of these criticisms highlighted perceptions of government-pharmaceutical industry collusion, with many participants discussing their perceptions of “Big Pharma” and how both the government and pharmaceutical companies work together to maximize profits over people’s wellbeing.

Below, Michael shares his perception that the U.S. government prioritizes capitalism over the wellbeing of regular people, giving the example of collusion with the pharmaceutical industry that produces profit but “never” the cure:

*I think a lot of bad people are elected*,* which is to say that a lot of people who either don’t understand science or don’t really have the good will of people at the top of their list… I don’t believe in free market capitalism because it doesn’t work. It doesn’t have people’s best interest in mind… if we’re not regulating these companies*,* like pharmaceutical companies*,* then what incentive do they have to actually help put an end to the disease that afflict us? To them*,* they make money every time they sell something that suppresses the symptoms but never is a cure.* (Michael, Asian non-Hispanic, 25, Prior PrEP use)

Similarly, Nic, quoted below, criticized the U.S.’ health insurance system, perceiving the pharmaceutical industry, health insurance companies and “CEOs” to benefit while “individuals” struggle to afford care:

*I think a lot of people have issue with just how much different medications cost and how*,* at least in the United States*,* our entire healthcare system seems to be set up to benefit huge pharmaceutical companies more than individuals*,* and insurance companies as well… unless someone has insurance*,* a refill on a prescription can cost thousands of dollars sometimes*,* even though the actual pills themselves don’t – obviously don’t cost that much. But the price just gets inflated to benefit these companies*,* and the CEOs of these companies*,* and insurance companies as well*,* for those who have insurance*,* rather than individual people who need medication or need other types of medical care.* (Nic, White Non-Hispanic, 25, Never used PrEP)

Anthony expressed that the system in the U.S. is “unrealistic” and that caring for people was “on nobody’s priority list”:

*Is PrEP affordable? I’ve never taken it. I don’t have health insurance. I would not know… I just think health insurance – the industry as a whole – is just too expensive. I just think it’s unrealistic… I don’t know. I try not to think about it. It really makes me sad… That it’s so inaccessible and I think it’s the easiest way to decrease cost is taking care of yourself*,* taking care of your people*,* and that is on nobody’s priority list I feel like.* (Anthony, Latine, 25, Never used PrEP)

#### Subtheme 2b. HIV Cure/Origin Ideas Reveal the Intersectional Nature of Structural Inequity

Most participants expressed significant mistrust of the pharmaceutical industry and/or government, and these sentiments were often manifested in the belief that there is already a cure for HIV, but it is being withheld for profit reasons. Some participants also believed that HIV was deliberately created for nefarious purposes, such as genocide of minoritized populations. While some participants endorsed these ideas, many other participants expressed that while they did not personally believe such notions, or were not certain, they felt it was highly possible and believable based on their understanding of the historical and ongoing injustices in the U.S. Nearly all participants discussed socioeconomic inequality as a driving force in American society. Participants also brought in dimensions of racial inequality, as well as homophobia. Although participants of color spoke of these issues in the context of their lived experiences, many White participants also mentioned structural racism as part of the landscape of inequity that characterizes American society.

Asked if a cure for HIV would be available to everyone, Marcus highlighted the class inequality dimensions:

*I believe [a cure for HIV] will only be available to certain people where if you’re rich and you have the money*,* you can afford it*,* they give it to you. If you’re poor*,* you’re not gonna get it. They’ll just give you the runaround… And we have homeless people on the street more than ever nowadays*,* and it’s like*,* what’s being done about that?* (Marcus, Black, 26, Never used PrEP)

Below, Malik raised the role of racism and homophobia in healthcare access both globally and in the U.S. specifically. He notes that “blue states” would facilitate better access to a theoretical HIV cure than “red states”, where Black populations and sexual minority men would specifically be de-prioritized.

…*it’s foolish to think that – once a cure is found – it will be available to everyone… Western countries will have it first… But dead last will be the African countries… Because by Western standards*,* those people aren’t very important. They’re not worth – they’re not of much value to human society in accordance with Western standards… Within the United States*,* I would think that some states will have better access to it… Some states will have better access to it than others*,* but I would say the blue states – the more liberal states – will have better distribution of the drugs while we have the red states they’re like sticking up their middle finger like*,* “Screw you. Die faggot*,* die.” That’s just my opinion and it would definitely affect the African American population in the south because they’re barely getting by with their state’s welfare programs.* (Malik, Black/Latine, 26, Prior PrEP use)

Tariq perceived HIV to be potentially government-introduced as a method of population control. Asked about the potential for a cure for HIV in his lifetime, Tariq felt that a cure would not be in the best interest of the government, and that “minorities” would not have easy access:

*It’s a government beneficial thing like “Okay*,* well*,* our world is becoming overpopulated*,* so we need some people to die now.” … I just think the world is getting overpopulated*,* so [HIV] is another outlet for it to slow down… I think minorities*,* we can’t afford it*,* to get [the cure]… But why are we not getting that? Because we don’t have the funds to get that same medication… Because I don’t have the resources… So*,* they give you what’s available for you and what you can afford*,* and that’s it. Like health insurance. They give you what you can afford and that’s it…* (Tariq, Black, 27, Prior PrEP use).

### Theme 3. PrEP and Healthcare Providers Not Necessarily Perceived as Nefarious; Mistrust Can Coexist with Support for PrEP

For many participants, their suspicions toward the government and/or “Big Pharma” formed the background against which they made decisions about what to put into their bodies, including PrEP. Although some participants’ mistrust was strong enough to dissuade them from using PrEP, many participants shared high levels of mistrust but chose to use PrEP anyway. Thus, while these sentiments were extremely common among this sample, endorsing such beliefs did not necessarily mean they were opposed to PrEP; indeed, some participants were actively using PrEP at the time of data collection. Related and importantly, participants largely did not include healthcare providers, particularly primary care providers, in this narrative of government-pharmaceutical collusion. While a few participants did name physicians as part of the scheme, most did not express this notion or explicitly reject it.

#### Subtheme 3a: PrEP is a Public Health Good and is Not Necessarily Perceived as a Predatory Pharmaceutical Product

Participants expressed a range of nuanced perspectives on PrEP, balancing skepticism toward the pharmaceutical industry with recognition of PrEP’s public health benefits. While some, like Ethan, voiced concerns about the lack of long-term research, they still supported PrEP’s role in HIV prevention. Others, including Logan and Adam, acknowledged deep mistrust of pharmaceutical companies—describing them as “evil”—yet continued to use or support PrEP, often citing its accessibility through free programs. Overall, participants distinguished PrEP from other medications, viewing it as a uniquely beneficial tool, even amid broader institutional mistrust.

Below, Ethan notes that while he is wary about “lack of long-term research”, he also thinks PrEP helps people protect themselves:

*I’m for it. I was never on it when I was single. And I don’t ever think I would just because I’m just skeptical about the lack of long-term research on it*,* even though I know that there’s a lot of convincing evidence that should make me think otherwise. But I think it’s great that so many people are on it and adhering to it and getting tested every three months and minimizing their risk of exposure or minimizing their risk of contraction…* (Ethan, White Non-Hispanic, 26, Never used PrEP).

Like many other participants, Frankie, quoted below, expressed frustration with the health insurance system in the U.S., but did not perceive PrEP to be in the category of medications that typically draw suspicion. Criticizing that *“PrEP is $400 million without insurance”*, he was asked if he thought some people might be hesitant to use PrEP because of negative feelings toward the pharmaceutical industry:

*Frankie: Probably.*

#### Interviewer: Have you seen that [suspicion] play out?

*Frankie: I haven’t*,* no*,* not in relation to PrEP*,* but in relation to other medications*,* absolutely yeah.* (Frankie, Latine, 26, Prior PrEP use)

Max, below, expressed that while he did not know the details around the company that makes PrEP, or their profit margins, he still felt PrEP was a clear positive:

*I don’t know who owns Truvada… The company who owns them might be “big pharma”. I don’t know*,* but at least for my perception*,* it’s good for just the general public. It’s beneficial. It’s the only drug that’ll [prevent HIV]… That is good for the public health. It’s not drug peddling…It’s not pushing for the companies own profit because it’s good for you*,* I guess. They don’t seem like they’re just doing it for the money. …they do give those coupons or whatever where it’s like you’ll get it for free. …I don’t know how much Truvada costs if you’re buying it off the shelf*,* so I can’t really say – I don’t know if they’re making a killing off of it.* (Max, Asian Non-Hispanic, 25, Never used PrEP)

Logan, quoted below, felt that the pharmaceutical industry is “notoriously evil” but this perception has not affected how he thinks about PrEP generally or his own PrEP use, noting that he currently accesses free PrEP:

*I feel like pharmaceutical companies are notoriously evil… I definitely do not trust them*,* and since I’ve been able to PrEP for free*,* I haven’t really – it hasn’t really impinged upon that perception*,* but I can see a scenario. I’m always afraid I’m gonna go in one day and they’re gonna be like*,* “Oh*,* it’s $3*,*000.” I don’t know what I’d do.* (Logan, White Non-Hispanic, 25, Currently using PrEP)

Like Logan above, Adam expressed that pharmaceutical companies are “pure evil”. When asked how he reconciles that perception with taking PrEP, he stated PrEP is a “miraculous thing”, but also pondered the possibility that PrEP manufacturers provide initial vouchers to cause dependence on their product. Adam ultimately concluded that “we need medicine”:

… *it’s very plausible that pharmaceutical people are putting out all of these vouchers and all these things to really get people in it and on it*,* not because of this genuine desire to better mankind*,* but because later down the line when everybody’s so used to taking it and they decide to revoke all of that*,* people will start having to pay for it. I feel like that’s likely… but you know*,* we need medicine.* (Adam, White Non-Hispanic, 26, Currently using PrEP)

#### Subtheme 3b: Healthcare Providers Mostly Not Viewed as Part of Government-Pharmaceutical Collusion

Most participants did not perceive healthcare providers to be part of the collusion scheme. Participants either rejected the notion that providers are part of the government-pharmaceutical collusion, or when asked directly, did not mention them as key figures in the scheme. Counter perspectives are also shared below, where a minority of participants thought that providers benefited from government-pharmaceutical greed.

Philip, quoted below, did not perceive healthcare providers as part of the collusion scheme, stating that, in his opinion, the scheme was driven by pharmaceutical manufacturers, not doctors who will prescribe what is necessary to treat you:

Interviewer: *Do you think it’s at the doctor level*,* or? –*.

Philip: *Oh*,* no. It’s the pharmaceutical companies that are creating these medications. The doctor is gonna prescribe you this is the best thing that they know that’s gonna treat this*,* and the doctors will try to talk to you about the side effects and*,* like*,* and all these other things they know*,* but they’re not standing there creating it.* (Philip, Asian, 25, Prior PrEP use)

Gabriel, when asked which actors profit from medications sold by the pharmaceutical industry, did not factor in healthcare providers, but pointed to those in power:

*Probably anyone connected to them- I think government. I’m sure Trump is in there somewhere. Yeah*,* anyone who’s connected to those companies or has ties or benefits from them being empowered*,* for lack of better words.* (Gabriel, Black/Latine, 26, Never used PrEP)

Below, Wes who recently lost his parent’s health insurance coverage, shared that his grievances with the healthcare system was primarily at the “system” level, noting the U.S.’ history of medical racism led him to have some suspicions of PrEP, which he initially perceived PrEP to be “exclusively marketed towards gay men”:

*I’ve only had really great experiences in healthcare because I’ve had – I had really good healthcare thanks to my parents’ [insurance]*,* unfortunately not longer… So*,* I’m having trouble with that. It’s more of the system and the execution of the whole thing. So*,* I’m not necessarily standing from my experience*,* but I’m standing against it in large. regarding PrEP*,* I feel very skeptical – in our current American standing*,* what’s happening here with the pharmaceutical industry taking advantage of dying people. And on so many levels and this PrEP – okay*,* so like I don’t want to name history*,* but other times in history– within the last 50*,* 100 years*,* where we’ve seen powers where have used medicine against people. We think about the Tustecgic – I can’t say that word right now [Tuskegee] … Now how convenient that this product [PrEP] is available?… – I’m now seeing advertisements now for [PrEP] being for… heteronormative relationships… but I feel like when it first came out*,* seemingly from my perspective*,* it’s exclusively marketed towards gay men.* (Wes, Black Non-Hispanic, 26, Never used PrEP)

Although most participants did not consider individual healthcare providers to be key actors in government-pharmaceutical collusion, some did express this sentiment. Below, Jamie states, *“doctors want you to be sick*,* lawyers want you to be in trouble”*, suggesting some providers have a role in over-medicating people:

*I do feel like [the healthcare system] is business and it has more to do with the approach as far as you know*,* doctors want you to be sick*,* lawyers want you to be in trouble. That type of thing… you know*,* if you’re healthy*,* [doctors] don’t have work… So*,* that’s kind of my view on the whole system*,* you know?… I can see both sides of the coin. Of course*,* I respect what they do as well*,* but I feel that there is in our society – we are simultaneously being fed all of these things*,* you know*,* literally and figuratively… All those legal drugs [medications]…I feel like it’s being marketed to us but it’s also being demonized…Doctors are there to administer the treatment to the people who*,* you know*,* maybe they don’t have a sense of you know*,* what’s too much for them as far as like eating or smoking*,* drinking*,* anything.* (Jamie, Black, 27, Never used PrEP)

Jimmy also perceived providers to be part of the problematic healthcare landscape, sharing that he felt his provider “shaming” and “pushing” him to go on PrEP, which further dissuaded him from trying it:

*I’m not on [PrEP]. That’s another thing my providers have kind of shamed me for not being on in the past. Again*,* I guess because of my distrust of the medical system and because of my own behaviors*,* I haven’t felt like it was something that I needed to take*,* but no shame for someone who chooses to take it… I mean*,* I think it’s still a relatively new drug being introduced on a very massive scale at the moment… I still think there are possible complications that we don’t know about it and just the fact that every time I see a provider and then they kind of give me this shaming talk of “Why aren’t you on PrEP? You should be on PrEP. You really need to be on PrEP.” And that kind of pushes me to reaffirm this idea that no*,* I don’t think I need to be on it.* (Jimmy, White Non-Hispanic, 25, Never used PrEP)

## Discussion

This study highlights that medical, structural, and institutional mistrust is multifaceted and is critical to understanding patients’ hesitations and lack of engagement with PrEP. Our findings broadly conceptualize mistrust as an intersectional, multi-level phenomenon, rather than an individual-level characteristic or psychosocial barrier as it is sometimes portrayed in the literature [1, 4]. Rather than an absence of trust, a structural framing of mistrust suggests harm has been done and/or is ongoing, necessitating action on the part of society to acknowledge and remedy [4]. The participant narratives presented here highlight the intersectional nature of structural inequality in the U.S., encompassing the healthcare system but also the government and society more broadly. In this sense, the mistrust described here is systemic and therefore must be addressed at the structural and institutional levels.

Although PrEP was FDA-approved in 2012 and is based on decades-long research on ART, our findings suggest many YSMM have significant concerns around PrEP-related side effects and potential long-term effects. The extant literature also highlights that SMM for whom PrEP may be beneficial may hold inaccurate beliefs about the severity of PrEP-related side effects [35]. This phenomenon is common among SMM, where known side effects generate enough hesitancy to change the individual’s decision [36]. While the participants in this study primarily discussed the daily oral PrEP pill, these concerns remain relevant to long-acting injectable (LAI) PrEP. Unlike a pill that an individual can choose to stop taking, LAI PrEP is an injection with effects that cannot be undone after administration [37]. Concern over side effects remains one of the first-order barriers to PrEP initiation.

Aligning closely with the theme of PrEP-related effects is the belief that PrEP as prevention is not a compelling reason to initiate treatment. Participants in this study questioned if preventing HIV was worth the risk of enduring potential side effects. In this sense, some participants expressed that taking PrEP does not align with their views on health, as they seemed to view medications as necessary for treating illness but not preventing disease. This is especially true for participants who were not currently sexually active, have infrequent sex, or are in a monogamous relationship. These findings align with previous studies that have found similar hesitation around initiating PrEP, specifically the dubious benefit of ingesting a medication with known side effects and perceived severe unknown side effects as a means to prevent a disease the participant does not have [38, 39]. While this phenomenon belies a lack of understanding of disease prevention, it does suggest people conduct individualized cost-benefit analyses to determine if the prevention benefits outweigh the risk of potential side effects.

Most participants expressed perceptions of government-pharmaceutical collusion, viewing the federal government and “Big Pharma” as relentlessly profit-driven to the point that regular communities cannot access what they need, such as affordable medication and healthcare. This nefarious government-pharmaceutical industry collusion is well documented in the literature [40, 41] and is more prevalent among Black [31, 42, 43] and Latine [44] communities in the U.S. In the study presented here, White participants also expressed similar sentiments, suggesting this manifestation of mistrust has intersectional dimensions beyond solely minoritized race and ethnicity. Previous quantitative research with our study’s broader sample documented group-based mistrust among White SMM participants [45], suggesting additional research is needed that specifically explores how various manifestations of mistrust among sexual minority White men be similar and distinct from SMM of color. Related, although this analysis focuses on mistrust of the pharmaceutical industry, future research should endeavor to explore trust and mistrust phenomena surrounding the burgeoning wellness industry. As of 2025, the wellness industry has surpassed the pharmaceutical in by trillions of dollars [46]. Although participant criticisms of the healthcare system and pharmaceutical industry reflect data indicating that people in the U.S. collectively owe at least $220 billion in medical debt [47], scholars have called for increased scrutiny of and research on “Big Wellness” [48], noting its predatory marketing strategies (e.g., preying on people’s fear and vulnerability), lack of regulation and lack of scientific evidence. These concerns are especially disturbing in light of the current administration’s institutionalized science denialism that also preys on fear and vulnerability [40, 49]. 

Some participants in this study endorsed a belief that the pharmaceutical industry and/or federal government is withholding a cure for HIV for profit reasons, and/or that HIV was deliberated created and/or “released” to harm minoritized communities. These HIV origin/cure beliefs, often referred to as “conspiracy beliefs” in the literature, illuminate the intersectional nature of structural inequity in the United States. Participants highlighted the classism that structures American society, as well as emphasized differential access experienced by “minorities”. Extant research also strongly indicates that experiences of structural racism, homophobia and stigma in the healthcare system, and within interactions with providers, contributes to PrEP-related mistrust among Black SMM [50]. This study complements this understanding by also capturing how these experiences are within the healthcare system but also form the backdrop of society more broadly.

Despite most participants expressing structural and institutional mistrust, this study also suggests that individuals can hold mistrust and still consider and even initiate PrEP use. Some participants linked being able to access PrEP as perhaps the reason why they did not necessarily view PrEP as solely a predatory pharmaceutical product. This observation highlights the importance of equity in treatment access in building trust. Related, most participants did not include healthcare providers as part of the collusion, particularly primary care clinicians. Previous research has found that racially and ethnically minoritized populations with HIV do not necessarily perceive clinicians, especially primary care providers, as part of the government-pharmaceutical collusion motivated by economic greed [19]. Around HIV prevention specifically, researchers have found that trust in primary care providers was over three times more likely to be willing to try PrEP [27]. Although the analysis presented here is focused on providers as potential actors in perceived government-pharmaceutical collusion, it is still important to acknowledge that the broad role of providers in PrEP uptake is critical. Among this study’s sample, previous research found that healthcare providers’ perceptions of their sexual risk were often conveyed in stigmatizing ways, alienating them from sharing more [51]. In a prior study of Latino SMM, researchers found that higher endorsement of group-based medical mistrust, encompassing perceptions and experiences with providers, was associated with lower odds of positive outcomes along the PrEP cascade [52]. The study presented here concludes that while healthcare providers are not necessarily implicated in pharmaceutical-government collusion, healthcare providers, especially primary care providers, play a pivotal role in making space for and hearing these concerns. To address both patient concerns and mistrust and misinformation among individuals who may benefit from PrEP, clinicians can provide medically accurate and concern-specific information to enable patients to make a fully informed decision about their HIV-related prevention options [53]. Thus, interventions aimed at primary care settings are worthwhile efforts to build trust and address mistrust around sexual health and biomedical HIV prevention.

The extant literature holds that “conspiracy beliefs”, a phenomenon under the umbrella of medical mistrust, can be a barrier to engaging in HIV care [23], but not necessarily [54]. In a study of socially and economically marginalized Black and brown people with HIV in New York City, many participants expressed deep-seated structural and institutional mistrust, including endorsing HIV origin/cure beliefs (i.e., “conspiracy beliefs”); still, they were resolved to manage their HIV with ART and close engagement with their provider [55]. Indeed, patient decision-making is nuanced and accounts for coping strategies to help patients navigate the complexities of both the healthcare system and systemic ‘-isms’ that pervade daily life. Furthermore, recent scholarship indicates that some manifestations of medical mistrust may best be conceptualized as a potential coping strategy that fosters resilience [56]. Further research is needed to untangle these phenomena, specifically in the context of PrEP, as most HIV origin/cure belief research has been conducted with people with HIV.

Together, these findings suggest that addressing structural inequities is paramount to begin earning the trust of minoritized communities. Peterson and colleagues found that institutional distrust, as shaped by dehumanization, lack of confidentiality, and other harm experiences in correctional settings, was a barrier to PrEP use among SMM [57]. Similarly, previous research has found that medical mistrust partially explains the association between police brutality and unmet needs for medical care [58] suggesting more research is needed to more fully understand how mistrust is created and perpetuated by structural and institutional oppression. Future research on medical and structural mistrust should examine how harm can be acknowledged and, if possible, rectified, and how trust can be earned. In doing so, mistrust, distrust, and trust can be more accurately measured and thus acted upon [59]. In a recent qualitative study of academic researchers’ perspectives on mistrust and Black communities’ underrepresentation in research, the authors found that researchers agreed that the onus to build trust is not on participants but instead on researchers, and more broadly, institutions [60]. Indeed, the authors and other scholars highlight the importance of community-partnered research, noting that institutions, not individuals, must drive change [4, 60, 61]. In particular, Newman offers a much-needed critique of the literature’s predominant focus on Black people’s perceptions of harm and health behaviors rather than on the harmful institutions themselves, arguing that decentering the white analytical lens is essential [17]. 

### Limitations

There are many limitations to acknowledge. First, the study was conducted in NYC, where participants largely have access to healthcare tailored to the needs of sexual and gender minority people. The availability of state and city-funded resources provides more low-cost or free sexual healthcare service access in NYC than compared to other regions of the U.S. Thus, YSMM residing in areas with less developed public health infrastructures likely face greater challenges while engaging in medical care. Second, while 41sexual minority men and two transgender and non-binary individuals assigned male at birth participated in this study, we discourage conflating these needs and experiences of these distinct populations. Transgender participants were not excluded from the sample to honor their time and experiences; however, there was not sufficient representation to draw any substantial conclusions about their healthcare needs as transgender people. Finally, participants were primarily asked about daily PrEP and did not broadly explore LAI or on-demand PrEP.

### Clinical Recommendations and Conclusions

This paper has several important implications for clinicians delivering HIV prevention services. First, clinicians need training on the most effective ways to frame the risks of PrEP in discussions with patients. Many participants in this study had the perception that PrEP would expose them to significant potential organ toxicity – such as risk for renal toxicity - or other unknown adverse effects. However, serious adverse events with the most commonly prescribed forms of HIV PrEP are rare [62]. Adverse events with oral PrEP are rare enough that some experts have suggested PrEP manufacturers consider pursuing over-the-counter labeling for this medication, as was recently achieved for progesterone-only oral contraception [63]. Additionally, oral PrEP with tenofovir disoproxil fumarate-emtricitabine has been a component of HIV treatment regimens for over 20 years, thus the perception that PrEP is untested or could have many unknown risks is not accurate. Similarly, LAI PrEP, such as cabotegravir, has demonstrated a high efficacy and safety profile in multiple clinical trials [64]. Multilevel communication efforts (e.g., PrEP campaigns, clinician training, provider discussions) should be scaled up to support YSMM’s understanding of PrEP’s safety profile to minimize fear and hesitation around taking PrEP.

While LAI PrEP was not FDA-approved at the time of this study and participants were largely asked about daily oral PrEP, these findings remain relevant as PrEP advancements continue to emerge. Although LAI PrEP is currently available, it often has limited accessibility due to factors such as high cost, clinic logistics, insurance coverage and restrictions, workflow inefficiencies, and greater trust in pills versus injections [65]. For many YSMM, who often experience significant health disparities [66], such as unstable housing, limited transportation, lack of insurance, and negative healthcare experiences, such frequent and time-sensitive visits can present significant barriers. While oral PrEP can be managed more flexibly through telehealth or pharmacy refills [67], LAI PrEP requires in-person clinic visits [68]. The Centers for Disease Control and Prevention (CDC) clinical guidance outlines a multi-step initiation process that amounts to at least six in-person visits during the first year, compared to fewer and often more flexible appointments for oral PrEP, which typically involves baseline labs followed by quarterly follow-ups [68]. Related, although LAI PrEP is often positioned as a solution for those who struggle with daily pill-taking, a critical clinical fact remains: oral PrEP remains part of the injectable regimen, as CDC guidelines recommend an optional oral lead-in phase and, in some cases, oral “bridging” when injections are missed or during planned discontinuation to maintain protection during the drug tail period [68]. In practice, LAI PrEP does not eliminate the need for pills but instead changes the timing and context in which they are used. For individuals who cannot or prefer not to take pills at all, or who face unstable access to medication, these oral phases can become a barrier to starting or continuing LAI PrEP. The hope that injectables remove the burden of adherence risks must consider the real-world complexity of implementation. For YSMM facing logistical challenges and healthcare and/or institutional mistrust, managing both oral and injectable components may lead to confusion, disengagement from care, or worsened health outcomes. Thus, while LAI PrEP may reduce the need for daily medication, it introduces new clinical and logistical demands.

These LAI clinical considerations suggest that future research must explore unintentional burden and potentially widened PrEP disparities and identify appropriate implementation strategies to overcome these challenges. A recent study found of YSMM who discontinued daily oral PrEP found limited awareness and low understanding of long-acting PrEP formulations, though participants were open to learning about and potentially using it [69]. Notably, despite discontinuing daily oral PrEP, participants still expressed a clear preference for the monthly oral pill followed by injectables then subdermal implants, although they did show greatest interest in options that required fewer clinic visit [69], and a potential self-injection option [70]. Further research is needed not only to assess openness to alternative long-acting PrEP formulations, but also to explore how factors like trust and the frequency of clinic visits influence willingness to initiate and sustain PrEP use. While PrEP is often compared to birth control, public health efforts to increase PrEP uptake, especially LAI formulations, should learn from the well-documented coercion around long-acting reversible contraception among minoritized women [71]. As the communities most in need of HIV prevention closely overlap with communities that have been historically and continuously marginalized, we must be careful not to see PrEP become, or become perceived, as a tool a tool of oppression and bodily control.

Given the increasingly available options for biomedical HIV prevention, clinicians also need to better understand how YSMM think about prevention when considering how to make recommendations to patients about PrEP, as in this study, many YSMM did not find preventing HIV to be a compelling reason to take daily PrEP. Participants’ worldview around prevention may be essential to address for those patients with existing mistrust. Clinicians would benefit from guidance on how to navigate conversations with patients who express mistrust towards the medical establishment, the pharmaceutical industry, and/or the government broadly. This training should be linked to discussions about historical and ongoing mistrust grounded in poor treatment of minoritized communities at multiple levels in society (e.g., community, healthcare system, carceral system). Conversations about the risks and benefits of effective treatments like PrEP may not resonate if these underlying issues of mistrust are not explicitly addressed.

## Conclusions

Medical, structural, and institutional mistrust around health and healthcare are on the rise [72]. At the time of this manuscript’s finalization in mid-2025, the U.S. government has taken unprecedented steps to dismantle key portions of public health infrastructure, including the National Institutes of Health (NIH) and CDC [73, 74], These changes have wreaked havoc on research and funding support broadly, but disproportionately and intentionally around structural racism and sexual and gender minority health [74, 75]. As the COVID-19 pandemic demonstrated, the public’s trust in government, public health crisis management, and the scientific establishment is volatile and fragile, with a recent study indicated a 26% decrease in public trust of CDC between 2020 and 2024 [72]. As public health infrastructure is continually weakened, various manifestations of mistrust, and their resultant health consequences, will continue to unfold.

Although the underlying etiology of different communities’ mistrust can differ based on historical and ongoing lived experiences [40], this study’s participants largely expressed frustration and sadness when discussing their own experiences of and observations around structural inequity in society, including historical and ongoing institutionalized racism and classism. For many participants who expressed deep institutional and structural suspicions, access to non-judgmental, affordable healthcare, including PrEP, appeared to help them engage in care despite this mistrust. As policy changes in 2025 move toward drastic funding reductions for Medicaid and other social safety nets [76], while simultaneously increasing funding for violent policing and unlawful detention of brown, Black, and immigrant people [77], it is likely that experiences and perceptions of inequality will deepen. Ultimately, our findings suggest that mistrust is a meaningful factor in how people live their daily lives and make decisions about their health; although the narratives presented here were gathered prior to the pandemic, participants’ concerns about government neglect and intentional harm remain profoundly relevant as systemic injustices in the U.S. escalate at an alarming pace.
